# Menstruation in an Infant

**Published:** 1846-06

**Authors:** 


12. *Menstruation in an Infant.*—By W. H. Whitmore, Esq., Surgeon, Cheltenham. Among the family of Mrs. M. was a female child, who, from a few days after birth, had the catamenia regularly, at periods of three weeks and two or three days, until she had attained the age of four years and some months, when she died, after an illness of forty-eight hours. She was attended by Dr. Christie, who for more than a year before her decease, had satisfied himself of the fact. The detailed particulars were communicated to me by Dr. Christie, by whose permission I had an opportunity of witnessing the examination of the body.

When laid out for dissection, its great development was very striking—equalling that of a girl 10 or 11 years of age. The mammæ were unusually large, the mons veneris collapsed but well covered with hair, the labia pudendi sparingly so, though of unusual size for a child.

She was of fair complexion; and her hair, which was of a dark brown colour, was very plentiful. In the absence of her periodical ailments, she would enter into all the amusements of children of her own age; but when she was indisposed, she was exceedingly reserved, and would withdraw from all her playful occupations. When interrogated by familiar acquaintances as to her reason for absenting herself on these occasions from the amusements of other children, she would answer that she was indisposed; but when the same question was proposed to her by those with whom she was not intimate, she would merely blush, without making any reply. There were other young females in the same family, but in them the function referred to manifested no irregularity.—*Northern Journal of Medicine* for July, 1845, in *Medical Examiner.*

